# Phenotyping Viral Reservoirs to Reveal HIV-1 Hiding Places

**DOI:** 10.1007/s11904-025-00723-6

**Published:** 2025-02-04

**Authors:** Wenxuan Chen, Ben Berkhout, Alexander O. Pasternak

**Affiliations:** Laboratory of Experimental Virology, Department of Medical Microbiology, Amsterdam UMC, University of Amsterdam, Room K3-113B, Meibergdreef 15, 1105 AZ Amsterdam, The Netherlands

**Keywords:** HIV-1 reservoir, HIV-1 cure, Phenotypic analysis, Reservoir marker, Multiomics, Single-cell analysis

## Abstract

**Purpose of Review:**

Despite suppressive antiretroviral therapy (ART), HIV-1 reservoirs persist in various cell types and tissues and reignite active replication if therapy is stopped. Persistence of the viral reservoirs in people with HIV-1 (PWH) is the main obstacle to achieving a cure. Identification and characterization of cellular and tissue HIV-1 reservoirs is thus central to the cure research. Here, we discuss emerging insights into the phenotype of HIV-1 reservoir cells.

**Recent Findings:**

HIV-1 persists in multiple tissues, anatomic locations, and cell types. Although contributions of different CD4 + T-cell subsets to the HIV-1 reservoir are not equal, all subsets harbor a part of the viral reservoir. A number of putative cellular markers of the HIV-1 reservoir have been proposed, such as immune checkpoint molecules, integrins, and pro-survival factors. CD32a expression was shown to be associated with a very prominent enrichment in HIV-1 DNA, although this finding has been challenged. Recent technological advances allow unbiased single-cell phenotypic analyses of cells harbouring total or intact HIV-1 proviruses.

**Summary:**

A number of phenotypic markers have been reported by several independent studies to be enriched on HIV-1 reservoir cells. Expression of some of these markers could be mechanistically linked to the reservoir persistence, as they could for instance shield the reservoir cells from the immune recognition or promote their survival. However, so far no single phenotypic marker, or combination of markers, can effectively distinguish HIV-infected from uninfected cells or identify all reservoir cells.

## Introduction

Combination antiretroviral therapy (ART) effectively suppresses HIV-1 replication and reduces the viral load in plasma to undetectable levels, significantly decreasing HIV-related morbidity and mortality [1]. However, ART is not curative and has to be taken lifelong because the therapy fails to eradicate latent HIV-1 reservoirs that reside in various cell types and tissues, ready to reignite active replication if therapy is stopped [2, 3]. The viral reservoirs usually decay so slowly that their lifelong persistence is guaranteed [4, 5]. This long-term persistence is due to integration of the viral genome into a host chromosome. The integrated provirus persists for the lifetime of the infected cell, and division of such cell provides each progeny cell with an identical proviral copy. The viral reservoirs persist mostly through cellular longevity and proliferation [6–11], although replenishment by low-level HIV-1 replication despite ART might also contribute [12–14].

The average frequency of infected cells among peripheral blood mononuclear cells (PBMCs) in people with HIV-1 (PWH) on long-term suppressive ART is on average approximately 1 in 2,000 [6, 13], although this number varies profoundly among PWH. However, this refers to the total frequency of cells that harbor HIV-1 proviruses, which in ART-treated PWH are mostly replication-defective (unable to cause rebound) due to large deletions, hypermutation, etc. [15–18]. The frequency of genetically intact proviruses in ART-treated PWH is estimated to be approximately tenfold lower than that of total proviruses [16, 17]. However, not all intact proviruses are rebound-competent in vivo as some are integrated in the chromatin context that is repressive for HIV-1 transcription and cannot be reactivated [19–21], and others can be vulnerable to the host immune response that controls rebound [22, 23].

Viral reservoirs are usually defined as cell types or anatomical sites that support long-term persistence of replication-competent virus [24]. However, some defective proviruses can transcribe viral RNA and translate viral proteins [25–28] and thus may contribute to chronic immune activation and inflammation that are frequently observed in ART-treated PWH [29–33]. Thus, a broader definition of the HIV-1 reservoir might include all transcription-competent, including genetically defective, proviruses. As the reservoirs form the major obstacle to achieving HIV-1 eradication or a long-term remission, revealing HIV-1 hiding places – identification and characterization of cellular and tissue reservoirs for the virus – is central to the cure research. Several comprehensive reviews have recently summarized our knowledge of the HIV-1 persistence [34–38]. Here, we discuss some emerging insights into the phenotype and anatomic location of cells that harbor HIV-1 reservoirs.

### Cell Types and Anatomical Sites

Understanding the cell types and anatomical sites that harbor persistent proviruses is crucial for the design of curative strategies. The main HIV-1 reservoir is thought to reside in resting memory CD4 + T cells in the peripheral blood and lymphoid tissue [39, 40]. However, in the recent years, it became clear that HIV-1 persists in multiple cell types with distinct biological features within diverse tissues. Studies of HIV-1 tissue persistence in living PWH have focused on those anatomical compartments that can be sampled through biopsy, such as gut, liver, lymph nodes (LNs), and lungs, on cerebrospinal fluid (CSF) that can be collected via lumbar puncture, or on testicular or adipose tissue that can be obtained prior to sex reassignment surgery or during elective visceral surgery, respectively. Persistently infected cells have been repeatedly found in all these tissues [41–46]. De Scheerder et al. performed single-genome proviral sequencing of a short HIV-1 subgenomic region (V1-V3 region of the viral *env* gene) from PBMCs, LN, gut-associated lymphoid tissue (GALT), and CSF of 11 PWH on long-term ART [47]. Phylogenetic comparison of these sequences with those derived from plasma after analytical treatment interruption (ATI) suggested that the source of viral rebound could be heterogeneous with regard to tissue and anatomic compartments and that the rebound is predominantly fuelled by genetically identical viral expansions. However, a subsequent study from the same group that used near-full-length (NFL) proviral sequencing determined that sequence matches between proviruses recovered before an ATI and viruses sampled from plasma during rebound are actually rare [48]. This confirmed the results of earlier studies, which did not find any overlap between sequences of replication-competent or genetically intact proviruses on ART with those obtained from the rebound viruses upon ART interruption [49–51]. This suggests that the reactivation potentials of individual proviruses may differ in vivo and ex vivo, possibly due to the immune-mediated selection of rebound viruses in vivo [22, 23].

Because the number of anatomical sites that can be analysed in living PWH is obviously limited, studies on HIV-1 persistence in deep tissues generally rely on autopsy material [52, 53]. A perimortem observational research cohort study, called the Last Gift, was conducted at the University of California San Diego with the goal of understanding the distribution of HIV throughout the human body [54]. This study enrolled PWH diagnosed with a terminal illness from a non-HIV condition. Participants consented to blood sampling antemortem and donated their bodies for rapid postmortem autopsy. This allowed Chaillon et al. to collect blood and tissues from 6 PWH, including 4 PWH on uninterrupted ART, across 28 anatomical compartments [55]. Using single-genome *env* sequencing, they demonstrated that various anatomical sources contribute to viral dissemination. Moreover, intact HIV-1 *env* DNA was found throughout all anatomic compartments analysed, indicating that HIV-1 persisted in all deep tissues. However, the subgenomic sequencing approach used in this study again did not allow to infer the clonality or full genetic intactness of the persistent proviruses. To fill this knowledge gap, Dufour et al. measured levels of HIV-1 DNA, HIV-1 RNA, and performed NFL proviral sequencing using samples from 15 tissues of two ART-suppressed PWH who donated their bodies to HIV-1 cure research [56]. HIV-1 DNA was detected in all tissues analysed, with the lowest levels measured in brain and testes and the highest levels detected in lymphoid tissues. HIV-1 RNA and intact HIV-1 DNA was also detected in multiple tissues, indicating the activity and genetic intactness of HIV-1 reservoir in tissue, although large differences in both properties were apparent between the participants.

On the cellular level, apart from the extensively studied reservoir in CD4 + T lymphocytes, also γδ T lymphocytes and myeloid cells (monocytes, tissue-resident (*e.g.* urethral) macrophages, and brain microglia) have been shown to contribute to the HIV-1 reservoirs [57–61]. Here it must be noted that although a number of studies have detected HIV-1 DNA or RNA in various tissues or cell types, only a few could demonstrate the presence of inducible replication-competent virus. Veenhuis et al. developed a monocyte-derived macrophage quantitative viral outgrowth assay (MDM-QVOA) and detected replication-competent HIV-1 in monocytes in half of the participants [59]. On average, MDMs had a 1 log_10_ lower HIV-1 DNA level compared with their CD4 + T-cell counterparts. This study also used a myeloid-adapted intact proviral DNA assay (IPDA) to demonstrate that intact HIV-1 genomes in monocytes were present in 40% of the participants. As circulating monocytes have a lifespan of days, these data suggest that these monocytes may be derived from hematopoietic progenitor cells in the bone marrow that harbor latent HIV-1 proviruses [62, 63]. Tang et al. isolated viable microglia from brain tissue after rapid autopsy in the Last Gift cohort participants and detected total and integrated HIV-1 DNA as well as cell-associated (CA) HIV-1 RNA in these cells [60]. Moreover, these brain microglia harboured inducible virus that was replication-competent in both myeloid cells and CD8-depleted PBMCs. In addition, Banga et al. recently demonstrated that LN dendritic cells (DCs) harbor HIV-1 integrated DNA as well as replication-competent virus that can be induced upon TLR7/8 stimulation, despite years of suppressive ART [64].

### CD4 + T-Cell Subsets

CD4 + T cells can be subdivided into subsets with different functional, proliferative, and survival capacities [65, 66]. Although HIV-1 can persist in multiple CD4 + T cell subsets, numerous studies have explored which CD4 + T cell subpopulations, such as memory cell subsets and functional polarization lineages, preferentially harbor HIV-1 proviruses, particularly genetically intact or replication-competent proviruses. Among the functional polarization lineages, which are defined by specific antimicrobial properties and cytokine secretion patterns, different studies have found total, integrated, or intact proviruses to be enriched in Th1 cells (including those that express antiapoptotic factors), Th17 cells, Th1/17 cells, T follicular helper (Tfh) cells (especially in LNs), as well as tissue-resident memory T cells [67–75]. However, all polarization lineages in PWH on ART have been shown to contain HIV-1 DNA.

With regard to the maturation/differentiation phenotypes, HIV-1 can persist in stem cell-like memory (T_SCM_), central (T_CM_), transitional (T_TM_), and effector (T_EM_) memory CD4 + T cells, in addition to naïve (T_N_) CD4 + cells. Initial studies reported that the less differentiated memory subsets, including T_SCM_ and T_CM_, are particularly enriched in total HIV-1 DNA during ART [6, 76]. However, subsequent reports provided a mixed picture. By NFL single-genome provirus sequencing, Hiener et al. determined the ranking order of the intact provirus frequency from lowest to highest as T_CM_ < T_N_ < T_TM_ < T_EM_ [17]. However, a subsequent study that used IPDA assay did not find differences in the frequencies of intact proviruses between these subsets [77]. Hiener et al. also observed expansions of genetically identical intact proviruses in T_TM_ and T_EM_ cells. This was confirmed by Gartner et al., who used single-cell T-cell receptor (TCR) sequencing in cells that expressed HIV-1 p24 protein upon ex vivo stimulation with PMA/ionomycin and found that expanded infected clonotypes were overrepresented in the most differentiated subsets (T_TM_ and T_EM_) [78]. Using the same technique, Pardons et al. found that p24 + cells from ART-suppressed PWH are enriched in total memory (CD45RA-), T_TM_, and T_EM_ cells and underrepresented in T_N_ and T_CM_ subsets [79]. It should be noted that proviruses harboured by cells that can be activated to produce p24 in ART-treated PWH have subsequently been found in a number of studies to be overwhelmingly defective, mostly containing major splice donor defects [80–83]. Also, activation of CD4 + cells to detect p24-producing cells in virally suppressed PWH likely results in alteration of the cellular phenotype.

Subsequently, two studies observed an inverse correlation between the infection frequency of the CD4 + T cells and their half-life, with a gradient T_N_—> T_SCM_—> T_CM_—> T_TM_—> T_EM_ from the lowest to the highest levels of total and integrated HIV-1 DNA and CA HIV-1 RNA, and exactly the same gradient from the longest to the shortest half-lives [84, 85]. Notably, the highest frequencies of infection were found in T_EM_ cells that have half-lives of 2–4 months, which are nowhere near that of the HIV-1 reservoir (44 months). Similar memory subset distribution of HIV-infected cells was described by Grau-Expósito et al., who used single-cell FISH-flow assay to detect HIV-1 RNA + cells, and later by Kulpa et al. for integrated HIV-1 DNA and by Gálvez et al. for total HIV-1 DNA, although the latter study did not find clear differences by subset on the HIV-1 transcriptional level [86–88]. Interestingly, Reeves et al. found that in all subsets except T_N_, the cellular turnover rate is roughly an order of magnitude faster than the rate of decay of HIV-infected cells, suggesting that cellular proliferation consistently creates new HIV-1 proviral copies and differentiation quickly passages HIV-1 through the CD4 + cell maturation subsets [89]. Taken together, these results are in line with clonal expansion of infected cells being the most prominent mechanism of HIV-1 persistence under ART and suggest that reservoir cells with a differentiated phenotype are the progeny of infected T_CM_ cells undergoing antigen-driven clonal expansion.

With regard to intact or replication-competent proviruses, however, the picture is more complex. While T_N_ cells clearly contain less total proviruses than memory cells, Zerbato et al. demonstrated that T_N_ cells harbor a large inducible HIV-1 reservoir and the ratios of intact to total proviruses in T_N_ and T_EM_ cells may be higher than in other subsets [90]. The mechanisms of reservoir persistence in T_N_ and T_EM_ cells likely differ as the lifespan of T_N_ cells is much longer, while the reservoir clonality in these cells is much lower, compared with memory cells [85, 91]. In addition, CA HIV-1 RNA levels are at least an order of magnitude lower in T_N_ than in memory cells, and HIV-1 RNA/DNA ratios are also lower [85, 91], suggesting that the contribution of transcriptional latency to the persistence of intact proviruses is likely lower for T_EM_ than for T_N_ cells. T_EM_ cells might thus support HIV-1 persistence by other mechanisms, such as resistance to immune surveillance [92, 93].

Apart from the genetic intactness, a provirus needs to be inducible in order to be replication-competent (capable of igniting viral rebound). Some differences in the latency-reversing agent (LRA)-driven reactivation capacities between the CD4 + cell memory subsets have been reported [94, 95], however no clear trends emerged. In the study of Grau-Expósito et al., the combination of romidepsin and ingenol induced the largest proportion of cells to transcribe HIV-1 across most CD4 + T-cell subsets, with the exception of T_SCM_ subset [94]. Kulpa et al. found that, among T_CM_, T_TM_, and T_EM_ memory cell subsets, the T_EM_ subset harboured the highest frequency of cells with inducible multiply spliced HIV-1 RNA, as measured by *tat/rev*-induced limiting dilution assay (TILDA) [87]. Moreover, T_CM_ cells that were triggered to differentiate into T_EM_ cells before exposure to LRAs produced higher numbers of p24 + cells upon stimulation [87].

Studies discussed above used HIV-1 RNA transcription or p24 protein production as surrogate measures of latency reversal, which is not equivalent to replication competence. Kwon et al. determined the frequencies of replication-competent proviruses among resting T_N_, T_CM_, T_TM_, and T_EM_ CD4 + cells using a multiple-stimulation viral outgrowth assay [77]. After one round of stimulation, T_EM_ and T_TM_ demonstrated higher absolute frequencies of infectious virus-producing cells than T_CM_ and T_N_, confirming the results of another study, which found that differentiation into T_EM_ CD4 + T cells results in a higher frequency of the inducible HIV-1 replication-competent reservoir [96]. However, when Kwon et al. calculated proviral inducibility by normalizing these absolute frequencies to the frequencies of cells with intact proviruses, no difference between the subsets was observed. Additional rounds of stimulation did not change this, and only an average of 1.7% of intact proviruses could be induced to release replication-competent virus across all subsets after four rounds of stimulation, although large person-to-person variability was observed [77]. It must be noted that this study used resting CD4 + cells, excluding cells that expressed activation markers such as HLA-DR. While activated cells expressing HLA-DR have been shown to substantially contribute to cells harbouring intact proviruses [97–99], it remains to be demonstrated that these cells fulfil the definition of the long-lived HIV-1 reservoir.

In summary, although differences between relative contributions of the different CD4 + T-cell subsets to the HIV-1 reservoir undoubtedly exist, all subsets have been shown to harbor a portion of the reservoir, complicating the design of a targeted approach for HIV-1 eradication based on T-cell subsets.

### Markers of HIV-1 Reservoir Cells

Absence of virus-encoded surface markers on the HIV-1 reservoir cells greatly complicates the search for a cure. Identification of host cell markers of the reservoir has therefore become the “Holy Grail” of HIV-1 cure research [100]. These markers could contribute to the cure research by expanding our understanding of the biology of viral persistence, enabling accurate measurement of the reservoirs to evaluate the efficacy of therapeutic interventions, and providing a “handle” for selective therapeutic targeting of the reservoirs. A number of potential cellular markers of HIV-1 reservoir have been proposed, including CD2, CD20, and CD30 [101–103]. These and other markers have been previously covered by a comprehensive review [104]. Here, we will briefly summarize some recent developments that concern potential markers of the viral reservoir.

In chronic infectious diseases, T cells gradually lose their function and become exhausted due to constant stimulation [105]. During this period, T cells show increased expression of inhibitory receptors, also known as immune checkpoint molecules (ICs), which impede the normal function of these cells by raising the barrier for activation. Key ICs include programmed cell death protein 1 (PD-1), cytotoxic T-lymphocyte associated protein 4 (CTLA-4), T cell immunoreceptor with Ig and ITIM domains (TIGIT), lymphocyte-activation gene 3 (LAG-3), and T-cell immunoglobulin and mucin-domain containing-3 (Tim-3). It was hypothesized that ICs, through their ability to inhibit T-cell activation, could favour HIV-1 latency during ART, hence CD4 + cells expressing ICs could be enriched for persistent HIV-1 in ART-treated PWH [106]. Indeed, Fromentin et al. demonstrated that CD4 + T cells co-expressing PD-1, TIGIT and LAG-3 were enriched for integrated proviruses (median of 8.2-fold compared to total CD4 + T cells) [106] and Pardons et al. reported that p24-producing cells from ART-suppressed PWH were enriched in cells expressing PD-1 and TIGIT [79].

More recently, it was shown that CD4 + cells co-expressing PD-1 and CTLA-4 were modestly (< twofold) but significantly enriched for total HIV-1 DNA, and at the same time were less inducible for multiply spliced HIV-1 RNA production, compared to double-negative cells [107]. In a subset of these participants, it was subsequently demonstrated that genetically intact proviruses were less frequent in CD4 + T-cells that expressed CTLA-4, and proviruses found within PD-1 + cells are more likely to have intact open reading frames for viral genes such as *tat*, *rev* and *nef* [108]. In line with these studies, HIV-1 reactivation from latency ex vivo in CD4 + T cells from ART-suppressed PWH was enhanced by a PD-1 blockade with the monoclonal antibody pembrolizumab [109], and in vivo intravenous administration of the same antibody in a cohort of PWH on ART resulted in a modest (1.32-fold) increase in CA HIV-1 unspliced (US) RNA and a 1.61-fold increase in US RNA/HIV-1 DNA ratio [110]. Similarly, administration of a combination of another anti-PD-1 antibody (nivolumab) plus anti-CTLA-4 antibody (ipilimumab) to PWH on ART in another clinical trial resulted in a 1.44-fold increase in US RNA levels [111]. In line with this, a combined blockade of CTLA-4 and PD-1 induced robust viral reactivation in ART-treated, SIV-infected rhesus macaques and reduced total and intact SIV DNA in their LNs, although this was still insufficient to control or delay viral rebound after ART interruption [112].

Apart from ICs, other markers have recently been shown to be associated with the HIV-1 reservoir. Pardons et al. observed that p24-producing cells from ART-suppressed PWH were enriched in cells expressing integrin α4β1 (very late antigen-4 or VLA-4), a dimer composed of CD49d (α4) and CD29 (β1), with 74% of p24 + cells expressing this integrin on their surface, compared to 35% for p24- cells [79]. Subsequently, Dufour et al. from the same group demonstrated that, although cells with intact proviruses constituted a small minority of the cells that expressed p24 upon stimulation, the vast majority (92%) of p24 + cells with intact HIV-1 genomes expressed VLA-4, and memory VLA-4 + cells harboured 27-fold higher frequencies of cells with replication-competent HIV-1 than their VLA-4- counterparts [82]. VLA-4 is an adhesion molecule that is involved in trafficking of immune cells to inflammatory sites and has recently been shown to increase the susceptibility of resting CD4 + cells to HIV-1 infection after in vitro coculture with endothelial cells that express VCAM-1, the ligand for VLA-4 [113]. Thus, it was hypothesized that VLA-4 expression on p24 + cells could mark recently infected cells [82].

An important conundrum that concerns HIV-1 reservoir markers is whether their expression is the cause or the consequence of HIV-1 infection or persistence of infected cells. Expression of these markers could be either upregulated upon HIV-1 infection, or cells that express these markers could be more susceptible to HIV-1 infection, more prone to establishment of the viral reservoir, or have a better selective advantage to persist over time. Kuo et al. demonstrated that CD4 + cells expressing OX40, a secondary co-stimulatory IC that regulates the expression of the apoptosis inhibitor BIRC5 (survivin), were enriched for total and clonally expanded HIV-1 proviruses [114]. Expression of another molecular target of OX40, anti-apoptotic (pro-survival) protein Bcl-2, has been shown to mark CD4 + cells with higher levels of intact HIV-1 proviruses, and pharmacological inhibition of Bcl-2 was necessary for ex vivo depletion of the viral reservoir by anti-HIV T cells [115]. These results suggest that the reservoir is constantly being reshaped and selected over time to persist in specific cellular subset(s) with intrinsic properties that benefit the reservoir persistence, such as expression of ICs or pro-survival factors that shift the balance from apoptosis to proliferation. However, the Dufour study observed that individual cells carrying clonally expanded identical proviruses display very diverse phenotypes, indicating that cellular proliferation contributes to phenotypic diversification of the HIV reservoir [82]. This observation confirmed the results of another recent study demonstrating that cells that harbor clonally expanded identical intact genomes display diverse gene expression profiles [116].

A number of studies used systems biology approaches that allow comprehensive gene and protein expression profiling of HIV-1 latently infected cells, in order to identify new markers of reservoir cells. Using a label-free liquid chromatography-tandem mass spectrometry analysis, Zhang et al. identified CD98 as a membrane protein with an increased expression in multiple HIV-1 latently infected cell lines and primary CD4 + T cells compared to uninfected cells [117]. CD98^high^ resting memory CD4 + T cells from ART-treated PWH harboured significantly higher levels of total HIV-1 DNA, CA HIV-1 RNA, and intact provirus than the CD98^low^ counterparts. Furthermore, CD98^high^ CD4 + T cells exhibited robust proliferative capacity as measured by increased expression of the proliferation marker Ki67, and significantly contributed to the clonal expansion of the HIV-1 reservoir as 26% of the *env* proviral sequences were clonal in CD98^high^ resting memory CD4 + cells, compared with 3% in the CD98^low^ counterparts. Moreover, expression levels of ICs PD-1 and TIGIT were significantly higher on CD98^high^ CD4 + cells than on CD98^low^ CD4 + cells from the PBMCs of ART-treated PWH [117]. Interestingly, an earlier study found that killer cell lectin-like receptor subfamily B, member 1 (KLRB1), also known as CD161, which is expressed by most natural killer (NK) cells and by a quarter of CD4 + T cells, where it is widely recognized as a marker of the Th17 lineage, is also associated with a higher clonality of HIV-1 *env* proviral sequences (33% vs. 9%), as well as with a significant enrichment for total (6.7-fold) and intact (13.1-fold) HIV-1 DNA and for the replication-competent proviruses (2.1-fold) [118].

Using a dual-reporter HIV-1 construct that enables isolation and purification of uninfected, productively infected, and latently infected primary CD4 + T cells, coupled to mass cytometry by time of flight (CyTOF) that measures 40 surface proteins to provide immunophenotypic profiles at the single-cell level, and NanoString hybridization and fluorescence- based digital counting technology that allows for simultaneous detection of multiple mRNA and protein targets, Sperber et al. identified ecto-5′-nucleotidase NT5E (CD73) that commonly serves to convert AMP to adenosine as a factor that could regulate HIV-1 latency [119]. Expression level of CD73 in the LNs of ART-suppressed PWH was 1.6-fold higher on HIV DNA + cells that on uninfected cells. It was proposed that CD73-rich microenvironments could preferentially harbor HIV-1 reservoirs due to local hypoxia and paracrine adenosine signalling effects. Indeed, CD73 was preferentially expressed in cells that were in the vicinity of HIV-1 DNA + cells in ART-suppressed PWH [119]. However, a separate study did not find any enrichment for HIV-1 DNA in CD73 + cells [120].

Using quantitative proteomics-based isobaric stable-isotope labelling and high-definition liquid chromatography hyphenated with mass spectrometry, Beliakova-Bethell et al. profiled the entire proteome of primary T cell samples from an in vitro model of HIV-1 latency (a total of 10,886 proteins) [121]. Antibodies against selected proteins CEACAM1 (CD66a) and Plexin-B2 enabled enrichment of latently infected cells from cell mixtures by an average of 5.8-fold, and in a single sample from an ART-treated individual, the enrichment for HIV-1 DNA upon staining for these two proteins was 10.2-fold [121].

### CD32 as a Proposed Marker of HIV-1 Reservoir

In summary, cells expressing markers discussed above undoubtedly contribute to HIV-1 persistence during ART, but few of these molecules have been associated with any substantial enrichment for total or intact HIV-1 DNA or replication-competent proviruses, let alone demonstrated to exclusively mark HIV-1 reservoir cells. This can explain the excitement in the HIV-1 cure field when Descours et al. reported that CD32a, also known as Fc gamma receptor IIa, the low-affinity receptor for the immunoglobulin G Fc fragment, is associated with a ~ 1,000-fold enrichment for HIV-1 DNA and marks ~ 50% of the reservoir cells [122]. However, the excitement soon turned into disappointment when several groups were unable to reproduce the high HIV-1 enrichment in CD32 + cells [123–127]. To understand the reasons for this controversy, we undertook a rigorous study, which revealed that high purity of the isolated CD32 + CD4 + T cells (an almost complete absence of contaminating non-T cells that abundantly express CD32) is a necessary prerequisite for scoring a high enrichment for HIV-1 DNA [128]. CD32 + cells are much more frequent among antigen-presenting cells (APCs) than among CD4 + T cells. Therefore, even if the residual APC contamination of CD4 + T cells is low in general, APCs will be disproportionally overrepresented in the CD32 + fraction, which can easily obscure the enrichment for HIV-1 DNA. Furthermore, as shown by several groups, certain cell-sorting strategies and/or settings might result in the isolation of T-B cell doublets or conjugates instead of *bona fide* CD32 + CD4 + T cells [124, 129]. Therefore, specific efforts are necessary to thoroughly deplete the CD32 + non-T cells prior to the measurements of HIV-1 levels in CD32 + CD4 + cells.

With this in mind, we developed a sequential magnetic cell sorting strategy to first purify CD4 + cells by two rounds of negative sorting and to subsequently purify CD32 + CD4 + cells by positive selection [128]. Using this strategy, we measured a very prominent enrichment for HIV-1 DNA in CD32 + CD4 + cells from peripheral blood of ART-treated PWH (average, 292-fold), confirming the original results of Descours et al. [128]. To characterize the CD32 + CD4 + cells further, we recently developed a novel cell purification strategy that combined magnetic sorting pre-purification of CD4 + T cells followed by double FACS sorting for CD32 + CD4 + cells, which was validated by imaging cytometry. This strategy resulted in isolation of a small (0.015% of CD4 + T cells) but pure cell population with a dim but homogeneous CD32 signal, which was spread over the cellular membrane, suggesting endogenous CD32 expression [130]. Importantly, when CD32 + CD4 + cells were isolated from peripheral blood of ART-treated PWH using this new method, we still measured high enrichment for HIV-1 DNA (average, 284-fold) [130].

Several recent reports explored the role of CD32 that is not endogenously expressed by CD4 + T cells. Adams et al. studied CD32 expression in tissues from HIV-infected humanized mice suppressed on ART and reported a significant but limited enrichment for total HIV-1 DNA in bone marrow- and spleen-derived CD32 + memory CD4 + T cells, as well as a ~ tenfold enrichment for HIV-1 translation-competent proviruses in spleen-derived CD32 + memory CD4 + T cells [131]. Interestingly, in this study, CD32 appeared to be mostly present on cell fragments, possibly platelets (as B cells and monocytes were excluded), that were externally attached to CD4 + T cells, rather than expressed endogenously by CD4 + cells, but some enrichment for HIV-1 in CD32 + cells was still observed. Another study has demonstrated some enrichment for HIV-1 DNA in T-B cell conjugates (CD4 + CD19 +), as compared to total CD4 + T cells, in PWH on ART, irrespective of CD32 expression [132]. In a recent elegant (mostly in vitro) study, Albanese et al. reported that surface receptors can be transferred from primary macrophages to CD4 + T cells via long (up to 100 µm) filamentous donor cell membrane nano-protrusions and identified CD32 as both driver and cargo of this specific type of trogocytotic transfer [133]. Importantly, macrophage-derived membrane patches transferred to resting CD4 + T cells rendered these cells susceptible to HIV-1 infection by serving as hotspots for virus binding [133]. Whether this process occurs in vivo in PWH, and whether CD4 + T cells that receive CD32-containing membrane patches from other cell types via this mechanism are indeed more susceptible to HIV-1 infection, remains to be determined. However, even if this turns out to be the case, it will still not provide a straightforward explanation for the observed enrichment for HIV-1 reservoir cells among CD32 + cells in ART-treated PWH, because the reservoir mainly persists by proliferation of infected cells and most reservoir cells in PWH on long-term ART have likely never “seen” the infecting virus. A more plausible explanation for the HIV-1 enrichment in CD32 + cells would be that CD32 is expressed on a specific subset of cells with enhanced proliferation/survival, which would constitute an ideal niche for the long-term persistence and expansion of the reservoir cells. Alternatively, CD32 expression could interfere with immune-mediated elimination of the reservoir cells. In line with this reasoning, Astorga-Gamaza et al. recently proposed an attractive model, in which CD32 might protect HIV-infected cells from NK cell-mediated antibody-dependent cell cytotoxicity [134].

### Single-Cell Multiomic Approaches to Phenotype the HIV-1 Reservoir

Until recently, phenotyping the HIV-1 reservoir has largely involved sorting cells based on predefined sets of surface proteins, followed by quantitating HIV-1 by different assays. Although these studies have provided many meaningful insights into HIV-1 persistence, they have been limited to the comparison of cell subsets differing in one or several markers and do not provide a comprehensive view of the phenotypic features of reservoir cells. An alternative approach is to isolate the reservoir cells from ART-treated PWH and characterize their phenotype, in comparison with uninfected cells. As straightforward as it might sound, isolating the reservoir cells from an infected individual for phenotyping is not an easy task, because infected cells in ART-treated PWH are extremely scarce, heterogeneous, and, with rare exceptions, do not express viral proteins unless stimulated. Assays such as RNAflow-FISH, HIV-flow, HIV-1 SortSeq, or latent cell capture (LURE), which are based on ex vivo cell stimulation with subsequent capture of cells expressing HIV-1 RNA, HIV-1 proteins p24 or Env, or a combination of viral RNA and protein, have been developed [79, 81, 86, 135–137], but cellular stimulation causes dramatic changes in the expression of a number of surface markers, as well as in the cellular transcriptome.

In an elegant study, Neidleman et al. [138] attempted to alleviate these issues by using an analytical method called predicted precursor as determined by single-cell linkage for distance estimation (PP-SLIDE), which can trace reactivated cells to their original state [139]. PP-SLIDE predicts the phenotypes of resting CD4 + T cells based on the phenotypes of ex vivo activated cells, which retain some of their original identity despite activation. CD4 + T cells isolated from ART-treated PWH were divided into two pools: one pool was used to generate the atlas of unstimulated cells and the other pool was stimulated with HIV-1 Gag + cells phenotyped by CyTOF. The CyTOF profiles of reactivated cells were subsequently matched against those of the unstimulated atlas cells and the unstimulated target cells were identified using a k-nearest neighbor (kNN) approach. This method requires an important assumption that for every cell expressing Gag upon stimulation, a phenotypically similar cell (termed “kNN latent cell”) was present in the atlas of unstimulated cells. Relative to total memory CD4 + cells, kNN latent cells consistently expressed higher levels of ICs (PD-1, CTLA-4, OX40, inducible T-cell co-stimulator (ICOS)), as well as markers of T cell activation (CD69, CD25, HLA-DR) and differentiation (T-bet, CRTH2, CCR6). However, quite prominent donor-to-donor variation in kNN cell phenotypes was observed, and the panels of 8–10 markers designed to enrich for HIV-1 reservoir cells were different between participants. Despite this, two universal panels could be designed, differing only by TIGIT expression, which enriched for the replication-competent virus in unrelated donors [138].

Multiple studies have detected CA HIV-1 RNA in the majority of peripheral blood and tissue samples from PWH on prolonged ART in the absence of ex vivo stimulation [140–145], and in the recent years there has been increased recognition of the biological importance of these active viral reservoirs [146–149]. A recent report that used multiplexed RNAflow-FISH explored the phenotypic properties of unstimulated cells harbouring transcriptionally active HIV-1 reservoirs and found them to be phenotypically diverse but still enriched in T_CM_ and cells expressing CCR6, HLA-DR, PD-1, and ICOS, and underrepresented in T_N_ [150]. Another study used single-cell expanded CRISPR-compatible cellular indexing of transcriptomes and epitopes by sequencing (ECCITE-seq), which captures surface protein expression, cellular transcriptome, HIV-1 RNA, and TCR sequence within the same single cell [151], for a multiomic profiling of the single infected cells in PWH before and after ART initiation, with and without stimulation [68]. The numbers of HIV RNA + cells obtained in the unstimulated samples, especially from the viral suppression time points (n = 9), were too limited for a robust analysis. Nevertheless, this study determined that T-cell clones containing HIV-1 RNA + cells were larger than those without infected cells and were predominantly cytotoxic T_EM_ Th1 CD4 + cells expressing Granzyme B (GZMB). Interestingly, HIV-1 RNA + GZMB + Th1 cells expressed higher levels of Serpin B9, a serine proteinase that prevents self-inflicted injury from lytic granules (such as granzyme B) that fall back to the cytotoxic cells that produce them [68]. Thus, Serpin B9 expression in HIV-infected cytotoxic CD4 + T cells could render these HIV-1 reservoir cells resistant to cytotoxic CD8 + T cell killing, conferring a selective advantage to these cells and driving their preferential persistence [152]. Alternatively, preferential survival of cytotoxic CD4 + T cells could be due to high expression of Bcl-2, an apoptosis inhibitor [68].

Remarkably, two other recent studies also found that HIV-1 reservoir cells were enriched among cytotoxic CD4 + T cells. Weymar et al. performed single-cell transcriptomic analysis of CD4 + T cells belonging to expanded clones that harboured intact HIV-1 proviruses and observed that most of these cells were T_EM_ enriched for expression of some (but not all) cytotoxic T-cell genes, such as CCL5 and granzymes A and K [116]. In line with this, Pardons et al. found that CD4 + T cells from ART-treated PWH that expressed p24 after stimulation with a lipid nanoparticle containing Tat mRNA (this activation protocol does not change the transcriptome of CD4 + T cells, enabling the characterization of reservoir cells in their near-native state) expressed higher levels of Granzyme A and CCL5 (but not Granzyme B) than p24- cells, on both transcript and protein levels [83]. Interestingly, we recently reported that pure CD32 + CD4 + T cells cluster in the memory T-cell compartment with cytotoxic gene and protein signatures (increased expression of Granzyme A/K and Perforin compared to CD32-CD4 + cells), which may possibly provide one clue for the high enrichment for the HIV-1 reservoir observed in CD32 + cells [130].

Phenotyping only the transcription-/translation-competent HIV-1 reservoir (those HIV-1 reservoir cells that transcribe viral RNA without stimulation or can be stimulated to produce viral proteins or transcripts ex vivo) is very informative about the mechanisms of persistence of this subset of the reservoir, but will miss the reservoir cells with transcriptionally silent proviruses, such as those integrated in the suppressive chromatin context associated with “deep latency” [21, 153]. Recent technological advances allowed several groups to perform single-cell phenotypic analyses of cells harbouring total or intact HIV-1 proviruses, without limiting the analysis to proviruses that can be expressed. One of such advanced methods is ATAC with select antigen profiling by sequencing (ASAP-seq), a tool to simultaneously profile accessible chromatin and protein levels in the same single cell [154]. ASAP-seq is a combination of a previously developed single-cell assay for transposase-accessible chromatin using sequencing (ATAC-seq), which is based on Tn5 transposase binding to open chromatin regions, with elements of another assay called cellular indexing of transcriptomes and epitopes by sequencing (CITE-seq). Importantly, compared to flow cytometry or CyTOF methods that can maximally analyse ~ 40 surface proteins due to fluorophore spectrum overlap or the number of isotopes, CITE-seq can score expression of > 100 surface proteins simultaneously, limited only by the antibody availability.

Wu et al. used ASAP-seq to profile expression of 154 proteins in HIV-infected cells from untreated and ART-treated PWH [155]. From four ART-treated participants (three of them sampled longitudinally), they could isolate 213 HIV-1 DNA + cells. No consistent predominately infected cell type was found, and no marker could uniquely identify HIV-1 + cells. However, expression levels of some markers, such as CCR5, PD-1, CD2, signalling lymphocytic activation molecule 1 (SLAMF1), integrin α4, and HLA-DR, were significantly higher in HIV-1 + as compared to HIV-1- cells [155]. Some of these markers, such as PD-1, CD2, and integrin α4, have been associated with an enrichment for HIV-1 reservoir cells in independent studies [82, 101, 106].

As ASAP-seq cannot make a distinction between cells that harbor transcriptionally active and transcriptionally silent proviruses, Wei et al. recently applied DOGMA-seq, a multiomic assay that provides combined information on chromatin accessibility, mRNA expression, and protein levels in the same cell [154], to identify the single-cell transcriptional landscapes and surface protein expression(156 proteins) of transcriptionally silent (HIV-1 DNA + RNA-) *vs.* active (HIV-1 RNA +) infected cells [156]. Again, the low numbers of infected cells obtained from the viral suppression time points (19 HIV-1 DNA + cells and 14 HIV-1 RNA + cells) precluded robust statistical analysis in ART-treated PWH, but several proteins such as CD2, SLAMF1, KLRB1, integrins αL and α4, and CCR5, that were significantly upregulated in HIV-1 RNA + cells in viremia, were also upregulated in HIV-1 RNA + cells in viral suppression. Most of these markers have been identified by previous studies. Interestingly, on the transcriptional level, cytotoxic markers such as granzymes A and K and KLRB1 were upregulated both in viremia and after viral suppression in HIV-1 DNA + cells [156].

The main limitation of methods based on ATAC-seq, such as ASAP-seq and DOGMA-seq, is their inability to capture HIV-1 proviruses that are integrated into heterochromatin regions inaccessible to Tn5 transposase, which limits HIV-1 DNA + cell detection and introduces a bias towards detection of proviruses integrated in open chromatin, likely disfavouring the “deep latent” proviruses. In addition, these methods cannot infer proviral genome intactness. Sun et al. developed an assay termed ‘phenotypic and proviral sequencing’ (PheP-seq), based on the commercially available Tapestri platform (MissionBio) [157]. This single-cell assay analyses the surface phenotype (53 proteins) of unperturbed cells harbouring intact HIV-1, where intactness was inferred based on the PCR amplification and next-generation sequencing of 18 HIV-1 short genomic regions, including two from the previously described IPDA assay [158], in the single cells. IPDA has been previously shown to identify proviruses with a high probability to be intact. In the PheP-seq study, a provirus was deemed intact if either at least 20 sequencing reads from the two IPDA amplicons were available, or a minimum of 15 proviral amplicons from other genomic regions were present.

From the peripheral blood of 5 PWH on long-term suppressive ART, this study could obtain a total of 193 cells with intact proviruses. Global phenotypic analysis revealed that cells harbouring intact proviruses exhibited distinct phenotypic properties, whereas cells with defective proviruses were phenotypically largely indistinguishable from uninfected cells. In particular, cells with intact proviruses were enriched in expression of several ICs (PD1, TIGIT, B- and T-lymphocyte attenuator (BTLA), 2B4, and KLRG1). Importantly, these cells also showed elevated expression of several ligands for inhibitory receptors expressed on immune effector cells, such as herpesvirus entry mediator (HVEM), poliovirus receptor (PVR), HLA-E, and PD-L1 (a ligand for PD-1). These ligands increase resistance of target cells to CD8 + T cell- or NK cell-mediated killing, therefore their expression on the reservoir cells could confer a selective advantage for HIV-1 persistence. In contrast, in LNs, cells with intact proviruses (n = 111) predominantly showed phenotypic features associated with apoptosis resistance and survival (CD127, CD44, CD28, CD49d) [157], suggesting a possible difference in the reservoir persistence mechanisms between peripheral blood and tissues.

## Conclusions

Despite the multitude of studies that performed phenotypic analysis of the reservoir cells, the cellular identity of the HIV-1 reservoirs remains an enigma and so far no single phenotypic marker, or even a combination of markers, can effectively distinguish HIV-infected from uninfected cells or identify all reservoir cells. This does not make therapeutic targeting of the reservoirs an easier task, as any targeting approach should obviously spare the uninfected cells. Still, HIV-1 persistence seems to be far from random as a number of studies from different groups that used different techniques repeatedly identified the same phenotypic markers that are enriched on reservoir cells (Table 1). These markers will likely play an important role in future studies aimed at understanding and eradicating the HIV-1 reservoirs. It is an attractive model that expression of these markers could be mechanistically linked to the reservoir persistence, *e.g.* that some of these markers could shield the reservoir cells from the immune recognition and others could inhibit apoptosis of the reservoir cells and thus promote their survival. However, whether these or other potential mechanisms are the driving force of HIV-1 persistence in ART-treated PWH remains to be demonstrated.

**Table 1:** Phenotypic markers enriched on HIV-infected cells, identified in two or more single-cell multiomic studies

Marker		Study			
**CD number**	**Name**	**PP-SLIDE (138)**	**ASAP-seq (155)**	**DOGMA-seq (156)**	**PheP-seq (157)**
CD2	LFA-2		+	+	
CD11a	Integrin αL		+	+	
CD25	IL2RA	+			+
CD38	ADPRC1			+	+
CD44				+	+
CD45	PTPRC			+	+
CD49d	Integrin α4		+	+	+
CD69		+			+
CD134	OX40	+			+
CD150	SLAMF1		+	+	
CD161	KLRB1			+	+
CD183	CXCR3			+	+
CD185	CXCR5	+			+
CD195	CCR5	+	+	+	+
CD196	CCR6	+		+	+
CD278	ICOS	+	+	+	
CD279	PD-1	+	+		+
	HLA-DR	+	+	+	+
	TIGIT	+			+

Although recent technological advances have dramatically increased the number of proteins, which expression can be assayed in the same single cell, we need to note that the outcome of a phenotyping study is still inherently limited by the predefined set of antibodies used in that study, and some important markers can still be missed. It also needs to be highlighted that extensive inter- and intrapersonal cellular heterogeneity of the HIV-1 reservoir frequently limits the robustness of the phenotypic analyses, especially when only limited numbers of participants can be included in a study, or limited cell numbers can be isolated. In general, the deeper and more extensive the analysis, the lower the numbers of participants that can be included, the lower the generalizability of the results. Additionally, the reservoir markers can differ between peripheral blood and tissues. The significance of tissue microenvironments for the biology of the HIV-1 reservoir is being increasingly recognized, as survival, proliferation, migration, and renewal capacity of HIV-infected cells in tissues are influenced by multiple tissue-specific parameters, which may be distinct from peripheral blood.

In summary, exploring the vast and varied phenotypic landscape of the HIV-1 reservoir is a daunting task, which nevertheless needs to be accomplished in order to bring us closer to the lasting cure.

## Key references

- Lian X, Seiger KW, Parsons EM, Gao C, Sun W, Gladkov GT, et al. Progressive transformation of the HIV-1 reservoir cell profile over two decades of antiviral therapy. Cell Host Microbe. 2023;31(1):83–96.e5.
  - This study found that following two decades of continuous ART, the integration site profile of intact HIV-1 proviruses is heavily biased toward heterochromatin locations, likely as a result of immune selection mechanisms; such proviruses are less transcriptionally active and, possibly, less rebound competent.
- Einkauf KB, Osborn MR, Gao C, Sun W, Sun X, Lian X, et al. Parallel analysis of transcription, integration, and sequence of single HIV-1 proviruses. Cell. 2022;185(2):266–82.e15.
  - This study assessed in parallel a number of virological parameters, including proviral genetic intactness, chromatin context of the integration site, and transcriptional activity at the single-proviral level. This study strongly suggests that transcriptionally active proviruses are selected against during prolonged ART and that large clones of infected cells may outcompete negative selection forces through elevated intrinsic proliferative activity.
- DeMarino C, Denniss J, Cowen M, Norato G, Dietrich DK, Henderson L, et al. HIV-1 RNA in extracellular vesicles is associated with neurocognitive outcomes. Nat Commun. 2024;15(1):4391.
  - This study detected several classes of HIV-1 RNA (initiated, elongated, polyadenylated, spliced) in extracellular vesicles derived from cerebrospinal fluid and serum of ART-suppressed PWH; moreover, the detectability of vesicle-associated HIV-1 RNA was associated with neurocognitive dysfunction, suggesting that extracellular vesicles can be used as a marker of HIV-1 reservoir activity during ART.
- Dufour C, Ruiz MJ, Pagliuzza A, Richard C, Shahid A, Fromentin R, et al. Near full-length HIV sequencing in multiple tissues collected postmortem reveals shared clonal expansions across distinct reservoirs during ART. Cell Rep. 2023;42(9):113053.
  - This study analysed multiple samples from two PWH who donated their bodies for HIV-1 cure research and detected HIV-1 DNA in all tissues, with large variations across anatomical compartments and between participants. Genetically intact proviruses were more often found in lymphoid organs and half of the HIV-1 genomes in tissues were clonally expanded and frequently located in distant compartments.
- Veenhuis RT, Abreu CM, Costa PAG, Ferreira EA, Ratliff J, Pohlenz L, et al. Monocyte-derived macrophages contain persistent latent HIV reservoirs. Nat Microbiol. 2023;8(5):833–44.
  - This study developed a human monocyte-derived macrophage quantitative viral outgrowth assay (MDM-QVOA) and detected replication-competent HIV-1 proviruses in monocytes in half of their participants (PWH on ART). They also found genetically intact proviruses in monocytes in 40% of the participants.
- Banga R, Procopio FA, Lana E, Gladkov GT, Roseto I, Parsons EM, et al. Lymph node dendritic cells harbor inducible replication-competent HIV despite years of suppressive ART. Cell Host Microbe. 2023;31(10):1714–31.e9.
  - This study demonstrated that lymph node dendritic cells containing inducible replication-competent HIV-1 can be detected after years of suppressive ART.
- Collora JA, Liu R, Pinto-Santini D, Ravindra N, Ganoza C, Lama JR, et al. Single-cell multiomics reveals persistence of HIV-1 in expanded cytotoxic T cell clones. Immunity. 2022;55(6):1013–31.e7.
  - Using single-cell ECCITE-seq, this study profiled 267 HIV-1 RNA + cells and 68 expanded HIV-1 RNA + T cell clones. HIV-1 was found to reside in large and persistent GZMB + cytotoxic Th1 effector memory CD4 + T cell clones. The study suggested that HIV-1-infected cytotoxic CD4 + T cells may evade cytotoxic CD8 + T cell killing through Seprin B9 degradation of granzyme B.
- Dufour C, Richard C, Pardons M, Massanella M, Ackaoui A, Murrell B, et al. Phenotypic characterization of single CD4 + T cells harboring genetically intact and inducible HIV genomes. Nat Commun. 2023;14(1):1115.
  - By combining the phenotypic analysis of HIV-infected cells with near full-length sequencing of their associated proviruses, this study demonstrated that individual cells carrying clonally expanded identical proviruses display very diverse phenotypes, indicating that cellular proliferation contributes to the phenotypic diversification of the HIV reservoir. This study also found that the few cells that harbor genetically intact and inducible viral genomes express higher levels of the integrin VLA-4 compared to uninfected cells or cells with defective proviruses, and that memory CD4 + T cells expressing high levels of VLA-4 are highly enriched in replication-competent HIV-1.
- Pardons M, Cole B, Lambrechts L, van Snippenberg W, Rutsaert S, Noppe Y, et al. Potent latency reversal by Tat RNA-containing nanoparticle enables multi-omic analysis of the HIV-1 reservoir. Nat Commun. 2023;14(1):8397.
  - This study used a lipid nanoparticle containing Tat mRNA to reactivate HIV-1 without altering the transcriptome of CD4 T cells and identified transcriptomic differences, conformed at the protein level, between infected cells carrying an inducible provirus and uninfected cells.
- Duette G, Hiener B, Morgan H, Mazur FG, Mathivanan V, Horsburgh BA, et al. The HIV-1 proviral landscape reveals that Nef contributes to HIV-1 persistence in effector memory CD4 + T cells. J Clin Invest. 2022;132(7): e154422.
  - This study provided evidence that intact HIV-1 proviruses persisting in effector memory CD4 + T cells may be better protected from the host immune surveillance than in other maturation subsets, which might be associated with superior Nef-mediated MHC-I downregulation in the effector memory cells relative to less mature CD4 + T-cell populations.
- Albanese M, Chen HR, Gapp M, Muenchhoff M, Yang HH, Peterhoff D, et al. Receptor transfer between immune cells by autoantibody-enhanced, CD32-driven trogocytosis is hijacked by HIV-1 to infect resting CD4 T cells. Cell Rep Med. 2024;5(4):101,483.
  - This in vitro study found that CD32 drives trogocytosis of membrane patches between immune cells and that this cell–cell transfer is markedly enhanced by autoantibodies. Transferred receptors confer cell migration and adhesion properties, and macrophage-derived membrane patches render resting CD4 + T cells susceptible to HIV-1 infection by serving as hotspots for the virus binding. HIV-1 could hijack this mechanism by triggering the generation of trogocytosis-promoting autoantibodies to gain access to the target cells.
- Dubé M, Tastet O, Dufour C, Sannier G, Brassard N, Delgado GG, et al. Spontaneous HIV expression during suppressive ART is associated with the magnitude and function of HIV-specific CD4 + and CD8 + T cells. Cell Host Microbe. 2023;31(9):1507–22.e5.
  - This study identified phenotypically diverse HIV-infected cells that spontaneously express viral RNA, and occasionally protein, during ART. Despite carrying defective proviruses, active reservoirs correlated with HIV-specific CD4 + and CD8 + T-cell responses. These results suggest that ongoing viral gene expression maintains HIV-specific immune responses during suppressive ART.
- Wu VH, Nordin JML, Nguyen S, Joy J, Mampe F, Del Rio Estrada PM, et al. Profound phenotypic and epigenetic heterogeneity of the HIV-1-infected CD4 + T cell reservoir. Nat Immunol. 2023;24(2):359–70.
  - This study developed a single-cell strategy to precisely define the unperturbed HIV-1 reservoir in ART-treated PWH via the presence of integrated accessible proviral DNA in concert with epigenetic and cell surface protein profiling. The findings revealed the extensive inter- and intrapersonal cellular heterogeneity of the HIV-1 reservoir.
- Wei Y, Davenport TC, Collora JA, Ma HK, Pinto-Santini D, Lama J, et al. Single-cell epigenetic, transcriptional, and protein profiling of latent and active HIV-1 reservoir revealed that IKZF3 promotes HIV-1 persistence. Immunity. 2023;56(11):2584–601.e7**.**
  - This study used single-cell multiomics to simultaneously capture transcription factor accessibility, transcriptome, surface proteins, HIV-1 DNA, and HIV-1 RNA in memory CD4 + T cells from PWH during viremia and after suppressive ART. The study identified single-cell epigenetic, transcriptional, and surface protein states of latent and transcriptionally active HIV-infected cells and cellular programs promoting HIV-1 persistence. The heterogeneous HIV-infected cells were found to be driven by interferon responses, cytotoxic T cell differentiation, AP-1- driven TNF responses, and apoptosis.
- Sun W, Gao C, Hartana CA, Osborn MR, Einkauf KB, Lian X, et al. Phenotypic signatures of immune selection in HIV-1 reservoir cells. Nature. 2023;614(7947):309–17.
  - This study used a single-cell, next-generation sequencing approach for the direct ex vivo phenotypic profiling of individual HIV-infected memory CD4 + T cells from peripheral blood and lymph nodes of PWH on long-term ART. Peripheral blood cells harbouring intact proviruses frequently demonstrated signatures of surface markers conferring increased resistance to immune-mediated killing, paired with elevated levels of expression of immune checkpoint markers likely to limit proviral gene transcription, whereas cells with intact HIV-1 from lymph nodes exhibited a phenotypic signature primarily characterized by upregulation of surface markers promoting cell survival.

## Data Availability

No datasets were generated or analysed during the current study.
